# Access to cancer medicines in Kenya: a scoping review of costs, financial toxicity, quality of life and policy impacts

**DOI:** 10.3332/ecancer.2026.2135

**Published:** 2026-05-28

**Authors:** James Onyuro Oketch, Daniel Ogungu Onguru, Stephen Asito Amolo

**Affiliations:** School of Health Sciences, Jaramogi Oginga Odinga University of Science and Technology, PO Box 210-40601, Bondo, Kenya

**Keywords:** cancer medicines, financial toxicity, quality of life, out-of-pocket costs, health policy, Kenya

## Abstract

**Background:**

Cancer, Kenya’s third leading cause of death, imposes severe health and economic burdens, through high treatment costs and limited availability of cancer medicines. However, the full evidence landscape is fragmented. In this scoping review, we synthesise evidence on access to cancer medicines, financial toxicity (FT), Quality of life (QoL) and health policy impacts for the top five cancers in Kenya.

**Methods:**

Following Preferred Reporting Items for Systematic Reviews and Meta-analysis; extension for Scoping Reviews guidelines, we searched PubMed, African Journals Online, Google Scholar and grey literature (January 2018–May 2025) for studies on Kenyan adults, focusing on breast, cervical, prostate, esophageal or colorectal cancers. Eligible studies reporting outcomes on medicine access, FT, QoL or National/Social health insurance fund/Authority (SHA) were included. Studies were screened using Rayyan software and data synthesised descriptively and thematically.

**Results:**

A total of 60 articles were included. About one-quarter addressed health policy (15, 25%), while others focused on multiple objectives (14, 23.3%), medicine access (12, 20%), FT (10, 16.7%) and QoL (9, 15%). Most studies focused on breast (63%) and cervical cancers (50%). Medicine affordability was poor, costing 3.15–162.42 days of minimum wage per chemotherapy cycle, exceeding the World Health Organisation threshold, particularly for regimens including Trastuzumab. Public facility availability was below 50%, with procurement delays (4–8 months) contributing to stockouts. Treatment costs for stage I–III cancers ranged from USD 1,340–1,542 in public versus 10,915–11,862 in private facilities. FT affected 20%–54% of households and treatment abandonment due to costs was reported in over half (53.8%) of patients. QoL was generally poor (median scores 41.99–53), associated with FT and late-stage diagnosis (71% stage III/IV). Insurance provided inadequate coverage, although SHA’s KES 400,000 cap showed potential to reduce costs despite underfunding and limited adoption of expert advice. Major evidence gaps included the scarcity of data regarding pricing and catastrophic health expenditure measures.

**Conclusion:**

High treatment costs, limited medicine availability and inadequate financial protection constrain access to cancer medicines in Kenya. Strengthening supply chains, expanding insurance coverage and improving measurement of FT are critical to inform policies aimed at improving equitable access to cancer treatment in Kenya.

## Background

Cancer presents a significant health and economic toll globally. In 2022, there were an estimated 19.9 million new cancer cases and 9.7 million deaths worldwide. In Kenya, cancer ranks as the third leading cause of death. It accounted for 44,726 new cases and 29,317 deaths in 2022. The disease burden is driven mainly by breast, cervical, prostate, colorectal and esophageal cancers [1].

Despite the growing burden, delivering timely and affordable cancer treatment in Kenya remains challenging. As in many low- and middle-income countries (LMICs), patients face high out-of-pocket (OOP) costs, limited availability of essential cancer medicines and incomplete financial risk protection [2–4]. These constraints expose patients and their families to substantial financial hardship following cancer diagnosis.

Financial toxicity (FT), is increasingly used to describe the economic consequences of cancer and its treatment. FT encompasses both the objective financial burden and subjective financial distress. Objective burden includes OOP payments for medical and non-medical costs, such as transport as well as indirect costs, including income loss and reduced productivity. Subjective financial distress reflects a patient’s perceived inability to cope with treatment-related expenses [5]. It includes concerns about future costs and the need to adopt cost-coping strategies such as delaying treatment, borrowing or selling assets. In LMICs, FT manifests as catastrophic health expenditure (CHE), commonly defined as health spending that exceeds 40% of a household’s non-food income. An estimated 13%–68% of cancer patients in LMICs experience CHE. This level of financial strain is associated with delayed care, treatment abandonment and poorer quality of life (QoL) [5, 6]. However, although individual studies document FT in particular settings or cancer types, comprehensive evidence describing its scale and drivers in Kenya remains fragmented.

Access to affordable, quality-assured cancer medicines is a core requirement for effective cancer control. This is particularly important for breast and cervical cancers, where early and sustained treatment significantly improves survival. The World Health Organisation (WHO) access to medicines framework identifies four key pillars: availability, affordability, accessibility and quality as essential for equitable health systems [7]. In Kenya, these pillars are inadequately met. Public sector availability of essential cancer medicine is estimated to average below 50%. Affordability is further constrained by high medicine prices and reliance on OOP payments. Weak supply chains contribute to recurrent stockouts and inconsistent treatment continuity [3, 8–10].These challenges are compounded by limited public financing for health. Kenya allocates approximately 3.5% of its gross domestic product (GDP) to health. This falls well below the WHO-recommended threshold of 6% [10, 11].As a result, the health system struggles to deliver equitable and continuous cancer care.

Recent health financing reforms have sought to address these gaps. In 2023, Kenya transitioned from the National Hospital Insurance Fund (NHIF) to a new insurance scheme called the Social Health Authority (SHA) as part of broader efforts towards universal health coverage (UHC) and improved financial risk protection. However, early implementation has faced several operational and governance challenges. These include underfunding, limited uptake of expert recommendations, weak political commitment and disruptions in service continuity for chronic conditions such as cancer [10, 12]. The National Cancer Control Strategy 2023–2027 acknowledges many of these systemic weaknesses but continues to face implementation constraints [13]. This is in part due to fragmented and incomplete evidence on medicine access, patient-level financial outcomes and performance of health financing mechanisms.

In this review, we map available data on cancer medicine access, FT, QoL and health policy impacts (including NHIF/SHA outcomes) among Kenyan adults with breast, cervical, prostate, colorectal or esophageal cancers. By consolidating evidence across economic, clinical and policy domains, this review addresses fragmentation in the existing literature. It examines how medicine access and health financing arrangements shape patient experiences, treatment continuity and outcomes. The evidence aims to identify knowledge gaps and inform health system reforms under the SHA. Ultimately, this review seeks to guide future research priorities and policy making to enhance medicine affordability, availability and financial risk protection for people living with cancer in Kenya and comparable LMIC settings.

## Methods

This scoping review followed the Preferred Reporting Items for Systematic Reviews and Meta-analysis; extension for Scoping Reviews (PRISMA-ScR) guidelines, adhering to the methodological framework of Arksey and O’Malley , refined by Levac *et al* [14, 15]. The protocol was registered with the Open Science Framework (OSF) (contact jamonyuro@gmail.com for draft protocol). The review followed five stages that guide how scoping reviews are done: 1) identifying the research question, 2) identifying relevant studies, 3) study selection, 4) charting the data and 5) collating, summarising and reporting results.

### Search strategy and selection criteria

The review addressed the primary research question ‘What evidence exists on access to essential cancer medicines, FT, QoL and health policy effectiveness among adult cancer patients in Kenya?’ For the purpose of this scoping review, OOP costs refer to direct payments for care such as medicines, diagnostics, travel; CHE is healthcare spending exceeding 40% of household non-food expenditure; and FT encompasses OOP costs, income loss and coping strategies such as borrowing and asset sales. The review focused on the top five most prevalent cancers in Kenya, breast, cervical, prostate, colorectal and esophageal cancers, to align with Kenya’s health policy priorities.

Searches were conducted on 31 May 2025 in PubMed, African Journals Online (AJOL) and Google Scholar, limited to English publications from January 2018 to May 2025. A comprehensive search strategy was used, combining Medical Subject Headings (MeSH) and keywords tailored to each database. Key terms included ‘cancer,’ ‘Kenya,’ ‘essential medicines,’ ‘FT,’ ‘financial burden,’ ‘economic hardship,’ ‘financial distress,’ ‘CHE,’ ‘QoL,’ ‘NHIF,’ ‘SHIF,’ ‘SHA’ and ‘UHC,’ combined using Boolean operators (Supplementary Table S1 shows a search strategy used in PubMed).

Grey literature was sourced from WHO, Kenya Ministry of Health, NHIF/SHA and Kenya National Cancer Control Program websites, with credibility assessed by institutional authority and author expertise. Google Scholar searches were stopped after three consecutive pages of irrelevant results. Additional studies were identified from reference lists, scanning of included studies through manual searches and institutional repositories (University of Nairobi Digital Repository). Embase, Medline, Scopus and Web of Science were excluded due to access constraints, mitigated by AJOL (which indexes multiple journals in Africa, enhancing coverage of regionally relevant open access publications) and comprehensive gray literature searches.

To assess the eligibility of studies, the scoping review followed the Population, Concept and Context (PCC) mnemonic (Supplementary Table S2). Studies were included if published between January 2018 and May 2025, in English, and included primary research or policy evaluations. Exclusions included non-Kenyan studies, editorials and abstracts without full text. All citations resulting from the searches were imported into the web-based software platform Rayyan. After removing duplicated citations, two reviewers independently screened titles and abstracts, followed by full texts review and any disagreements on final inclusion were reached by consensus.

### Data analysis

Data were extracted from selected articles using an Excel form, piloted on six included studies (two quantitative, two qualitative and two mixed-methods) and refined accordingly. The data charting form captured study characteristics (author, year, design, location and publication type), population (cancer type, demographics and sample size), medicine access (prices, stockout rates and affordability), FT (OOP costs, CHE and coping strategies), QoL (scores, qualitative themes) and policy outcomes (NHIF/SHIF coverage, procurement and barriers) and main findings (Supplementary Table S3). Authors were contacted for clarification where necessary, such as cost data, with two responses received. Medicine availability was stratified by data source (national surveys, single-facility studies and supplier/agency assessments) using the Kenya Essential Medicines List (KEML) and WHO/HAI methodology, and data gaps and methodological differences documented.

We used the Mixed Methods Appraisal Tool (MMAT v. 2018) to assess the quality of studies given the different types of study designs included in the scoping review. The tool included methodological quality of quantitative studies (sampling, measurement, bias), qualitative (approach, coherence), mixed-methods (integration) and policy documents (clarity, relevance). Quality ratings informed interpretation but did not affect inclusion, as the scoping review’s objective was to map all relevant evidence.

Results are synthesised descriptively with numerical summaries of sample sizes and cancer types; and thematically by objectives (medicine access, FT, QoL and policy effectiveness), to show the extent and nature of evidence. Any other emerging themes relevant to the research question are reported. Where appropriate, we visualised findings using tables and graphs. Finally, the implications of findings, the broader context and recommendations for health system improvement and future studies are presented.

## Results

**Figure 1.**
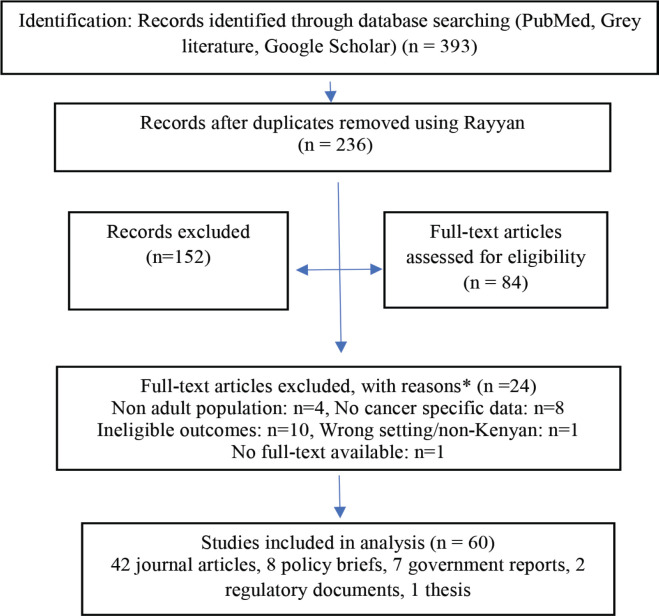
PRISMA-ScR flow diagram.

Following PRISMA-ScR guidelines, 393 articles were identified through searches in PubMed, AJOL, Google Scholar and grey literature (WHO, Kenya Ministry of Health, NHIF/SHIF and Kenya National Cancer Control Programme) conducted up to May 31, 2025. After removing 157 duplicates, 236 records were screened by title/abstract, with 152 excluded due to non-Kenyan settings (*n* = 80), non-cancer-specific focus (*n* = 42), editorials (*n* = 20) or abstracts without full text (*n* = 10). Full-text review of 84 records resulted in 60 included studies (42 journal articles, 8 government reports, 7 policy briefs, 2 regulatory documents, 1 thesis). Figure 1 presents the PRISMA-ScR flow diagram.

**Supplementary Figure S1.**
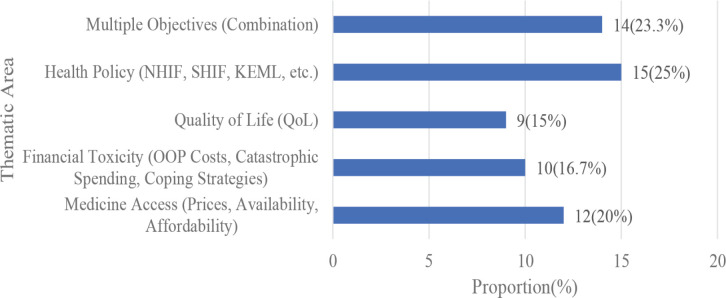
Distribution of articles by thematic area

About a quarter of articles were health policy related (15, 25%), followed by those touching on multiple objectives (14, 23.3%), medicine access (12, 20 %), FT (10, 16.7%) and lastly QoL (9,15%) (Supplementary Figure S1).

The quality of studies was assessed using the MMAT version 2018. The assessment excluded 13 non-research policy/guideline documents, hence included 47 studies. All had clear research questions and data addressing the research questions. The majority were quantitative descriptive studies (*n* = 31) and they generally performed well across MMAT criteria. All had a relevant sampling strategy (100%), appropriate measurements (100%) and appropriate statistical methods (94%). However, representativeness of the sample (68%) and low nonresponse bias (78%) were somewhat lower. Qualitative studies (*n* = 19) showed moderate adherence to MMAT criteria, with 74% meeting standards for an appropriate qualitative approach. There were 14 mixed-methods studies, which had an adequate rationale for mixed methods (71%) and effective integration of components (86%), though adequate interpretation of integrated outputs (64%) and addressing divergences between quantitative and qualitative results (57%) were lower Table 1.

Most studies were conducted in urban settings (Nairobi 28, 47%, Eldoret 10, 17%, Kisumu 8, 13%, Mombasa 6, 10%), with only 6/60 (10%) reporting rural data. Studies primarily focused on breast (38, 63%), cervical (30, 50%), prostate (16, 27%), esophageal (11, 18%) and colorectal cancers (9, 15%), with some addressing multiple cancers. Sample sizes ranged from 2 to 37,500 (median 151, IQR 77–334), with participants predominantly female (72%), aged 40–68.5 years and of low-to-middle socioeconomic status (77% unemployed, household income <KES 5,000/month) Table 2 summarises key study characteristics.

### Synthesis of results

The primary assessment focused on access to essential cancer medicines (prices, availability, affordability), FT, QoL and health policy effectiveness. No study investigated all four interconnected aspects in a unified manner. The focus of the studies varied from access and affordability to FT and its coping mechanisms; from QoL assessments to health policy evaluations and their implementation barriers, with some studies addressing multiple outcomes. The findings are organised thematically into the four outcomes.

About 12 studies (19%) reported medicine access using the WHO/HAI methodology or KEML assessments. Pricing data showed high costs. One study showed the minimum and maximum medicine prices across providers [3]. Minimum prices ranged from KES 140 for Methotrexate to KES 176,000 for Pembrolizumab 100 mg [3].Another study reported specific pricing, with public sector costs for treating stages I–III of breast and cervical cancers ranging from $1,340 to $1,543 and private sector costs significantly higher at $7,500–$11,862 [45].General chemotherapy costs ranged from KES 6,000–600,000 per course [8].Targeted therapies like Trastuzumab required 69.67–151.74 days of minimum wage per cycle [3].

Medicine availability varied significantly by facility type across the studies. National facility surveys, such as the Kenya Health Facility Assessment [57, 59], reported an overall availability of 44% for 24 tracer medicines (*n* = 2,896 facilities). Single-facility studies indicated higher availability in tertiary hospitals (50.8%, *n* = 4 hospitals) for drugs like Carboplatin (78.9%) and Trastuzumab (73.7%). Supplier and agency assessments reported lower availability at supply agencies, 33.5%(*n* = 5) for tier 1 suppliers and 40.2% (*n* = 8) for tier 2 suppliers, with generics more available (46.9%) than originator brands (6.7%) [3]. Notably, 78.3% of studies (47/60) lacked specific availability data, particularly for private facilities.

Affordability was poor, with all medicines exceeding the WHO affordability threshold of 1 day’s wage, rendering them unaffordable. Treatments involving biological therapies such as Trastuzumab were significantly more expensive, requiring several months of minimum wage. One cycle of chemotherapy involving Doxorubicin/Cyclophosphamide (HER2 negative) required 3.15–9.69 days of minimum wage; Cyclophosphamide then Paclitaxel/Trastuzumab (HER2 positive) required 78.36–162.42 days; Trastuzumab, 69.67–151.74 days; Docetaxel, 9.34–10.28 days [3]. The cheapest supplier and the cheapest private hospital often provided lower costs compared to Kenyatta National Hospital, the main cancer referral hospital in Kenya, particularly for expensive regimens involving Trastuzumab (Supplementary Table S4) [3] Table 3.

FT was reported in 10/60 studies (17%), with limited available data suggesting OOP costs ranging from $1,298 to $12,713/year [46]. Public sector costs for stages I–III breast and cervical cancers were $1,340–$1,543, while private sector costs were $7,500–$11,862 [45].Cervical cancer patients faced OOP costs of 87% for medication, 84% for travel and 75% for diagnostics [16]. OOP constituted 24% of total health expenditure (KSh 108B in 2020/21), with limited NHIF uptake (17% population, 27% informal sector) exacerbating the burden [10].CHE affected 20.27%–54% of households [38, 43].Coping mechanisms included borrowing (81%), selling assets (73%) and seeking charity (13%) or family/church support (10%) [16].Treatment abandonment was noted, with 53.8% of breast cancer patients forgoing care due to costs [4].However, the review noted sparse data and wide OOP variation, disadvantaging comprehensive/definitive affordability assessments/ population-level projections, as only 10 studies covered specific OOP or CHE data (Supplementary Table S5).

About 9/60 studies (15%) reported QoL, indicating compromised QoL, with median global health status scores of 41.99–53 (EORTC QLQ-C30, FACT-Cx). Breast cancer patients reported 64% good QoL, prostate 85% [20, 22]. Advanced disease (AOR = 7.3, *p* < 0.0001) and comorbidities (OR = 3.1, *p* = 0.037) predicted poor QoL [20]. High symptom burdens included fatigue (56%), pain (65%) and financial difficulties (79%) [30].Some studies reported thematic findings without quantitative tools.

Qualitative themes from ten studies highlighted psychosocial distress, stigma, social isolation, and physical limitations. In the physical domain, symptom burden and functional limitations were key, including fatigue, pain, weakness, dry mouth, insomnia/hypersomnia and mobility/self-care difficulties. Cervical cancer patients reported poor physical (60%) and emotional QoL, with hygiene challenges and family dependence. Multi-cancer studies noted reduced occupational functioning [17, 26, 30, 31]Better QoL was associated with early-stage disease, urban residence and stable remission [20]

Psychological themes included depression (59.4% in breast cancer), influenced by late-stage diagnosis (OR = 1.61, *p* = 0.319), employment (OR = 3.7, *p* = 0.058) and chemotherapy (neoadjuvant OR = 9.43, palliative OR = 9.5, *p* < 0.05), leading to reduced mental resilience and ‘constant worry’ about survival/family [24, 31].

Social domain themes featured isolation, stigma and family burden, with avoidance/discrimination (awkwardness: 2.51 ± 0.75; severity: 3.22 ± 1.29). Breast/cervical patients faced rejection/over-dependence, strained relationships, reduced social life, ‘community stigma’ and policy-level discrimination (2.99 ± 1.17), intersecting with financial strain via borrowing/charity [18, 31, 34]Spiritual themes showed religion as hope/coping, but unmet needs worsened distress in metastatic cases [18, 31]. Healthcare/systemic domain included unmet information needs (low cancer knowledge: 23.6%), system delays and financial difficulties (79%), amplifying access issues [20, 30](Supplementary Table S6).

About 15/60 studies (25%) evaluated health policy effectiveness, focusing on NHIF, SHA and the National Cancer Control Strategy (2023–2027). Stratifying by policy era, 13/15 policy studies (87%) focused on pre-2023 NHIF implementation, reporting low NHIF uptake (9%–17%) primarily covering inpatient services, and limited impact on treatment affordability, covering only 4 of 8 chemotherapy cycles [16, 62]. Post-2023 SHA data (limited to 2/60 studies) showed potential to reduce OOP costs via mandatory contributions (2.75% income-based) and the Emergency, Chronic and Critical Illness Fund (ECCIF), covering oncology services (chemotherapy KES 5,000/session, 1st line treatment limit KES 400,000 from SHA, KES 250,000 from ECCIF), but faced infrastructure and funding gaps [62, 68]No studies directly compared pre- and pos insurance policy transition outcomes. Health financing remained limited at 3.5% of GDP (below WHO’s 6%), with OOP costs at 24% of total health expenditure (KSh 108B in 2020/21), and weak cancer registries (64% missing staging data) hindered progress [11, 62, 69] Decentralisation efforts aimed to improve access, but oncology services remained urban-centric, with limited screening/treatment capacity in rural areas [23, 39] A medium-term expenditure report highlighted Kenya Medical Supplies Agency (KEMSA’s) role in procuring essential cancer medicines, such as chemotherapy, hormonal therapies, aligned with WHO EML, but noted barriers, including stockouts, inadequate procurement and urban bias [62]. Another study reported NHIF/SHIF schemes and UHC implementation, but identified barriers such as limited NHIF uptake (17% population, 27% informal sector) and infrastructure/capacity limits [12] (Supplementary Table S7).

From the reviewed documents, 8/60 studies identified the need to expand NHIF/SHA coverage to reduce OOP costs and FT. About 3/60 studies identified the need to strengthen KEMSA. Other policy suggestions included subsidising costs (3/60), enhancing early detection and screening programs (7/60), integrating psychological and QoL support (5/60), strengthening health infrastructure and workforce (4/60), enhancing cancer registries (2/60), monitoring and management of treatment adverse effects (3/60) and promoting digital and community support to provide education and psychological care (1/60) Table 4.

## Discussion

This scoping review of 60 articles published between 2018 and May 2025 mapped the nature and distribution of evidence on cancer medicines access, FT, QoL and health policy performance for breast, cervical, prostate, colorectal and esophageal cancers in Kenya. Across the literature, three patterns consistently emerged: high medicine costs, low public sector availability and inadequate financial protection through health insurance. Our review builds on and extends prior literature by consolidating fragmented evidence across systems of access, financing, QoL and policy interactions with a focus on Kenyan context.

The high cost and low availability of cancer medicines align with broader trends reported across LMICs. Yet, several gaps within Kenya are evident. Procurement delays of 4–8 months at the KEMSA were frequently reported and translated into medicine stock outs at public facilities. Public-sector availability remained below 50% across studies, indicating persistent supply constraints. In contrast, Uganda achieved 85.8% availability, exceeding the WHO’s 80% target, facilitated by centralised procurement [70] Compared to a study that mapped African medicine access methods [71], this review offers detailed insights on cost and policy effectiveness. Chemotherapy regimens such as Doxorubicin/Cyclophosphamide required 3.15–9.69 days of minimum wage, while targeted therapies like Trastuzumab required 69.67–151.74 days of minimum wageexceeding WHO affordability thresholds [3, 8, 44]. This renders guideline-recommended care inaccessible for most patients. Public-private sector disparities, indicating treatment costs as higher in private facilities, an eightfold increase in some cases, further deepen inequities. With about 73% of Kenyans reliant on the public sector, these differences restrict continuity of care and erode patient survival [39].

The review notes scarcity of data for colorectal and esophageal cancers (addressed in only 4 studies), limiting cost-effectiveness analyses for high-mortality cancers, despite rising incidence.

FT emerged as the most consistently documented patient-level consequence across included studies. OOP payments, ranging from US$1,298 to US$12,713 annually, placed substantial pressure on households. About 20.3%–54% of households experienced FT and elevated risks of CHE. Medication costs (87%), transport (84%) and diagnostic services (75%) were major cost drivers contributing to this burden [4, 16, 43, 63]. Patients frequently adopted severe coping strategies, including borrowing (81%), asset sales (73%), fundraising, and treatment abandonment (53.8%). Such patterns reflect deep financial strain upon cancer diagnosis. A systematic review reported a pooled CHE prevalence of 43.3% (95% CI 36.7–50.1) among cancer patients globally [72].However, the wide variation in OOP estimates due to inconsistent reporting approaches, diverse CHE definition, and heterogeneous samples (different cancers, stages and time points) limits comparability. Most studies were cross-sectional and descriptivelimiting restricting insights into how FT evolves across treatment trajectories. In addition, most studies lacked OOP payment and CHE data, limiting comparability. Similar methodological concerns have been noted in previous systematic reviews [73, 74].Importantly, validated FT tools like COST, remain underused in Kenya, limiting robust comparative evaluation of financial burden. These tools (COST-FACIT, PROFFIT) offer standardised, patient-centered assessment of both objective and subjective financial distress [75].

QoL outcomes were reported in 9 of the 60 articles, indicating limited integration of patient-reported outcomes in Kenyan oncology research. Global health scores measured using the EORTC QLQ-C30 ranged from 41.99 to 53, indicating moderate to poor overall functioning. Most studies particularly covered breast and cervical cancers. High symptom burden contributed substantially to poorer QoL. Reported symptoms included fatigue (56%), pain (65%) and financial difficulties (79%). Psychosocial distress was also prevalent, with depression ranging from 43.9% to 59.4% [20, 30, 76]. Qualitative findings further highlighted psychosocial impacts, including stigma, anxiety, body image concerns and social isolation [4, 16, 31, 46]

Clinical factors also influenced QoL outcomes. Early disease stage and stable treatment response were associated with better QoL. One study reported seven-fold higher odds of good QoL among early-stage cervical cancer patients (adjusted odds ratio = 7.3). However, the variation in assessment tools (EORTC QLQ-C30, FACT-B, HAM-D, Functional Evaluation of Chronic Illness Therapy) undermined cross study comparability. Most studies were cross-sectional and few examined longitudinal changes in QoL during the cancer care continuum. Longitudinal research is therefore needed to capture interaction between FT, treatment progression and patient-reported outcomes overtime. A previous study conducted in LMICs demonstrates the value of standardised metrics and reports robust QoL data, although covering only breast cancer [77]

At the policy level, the evidence base revealed persistent gaps between policy design and the level of financial protection experienced by cancer patients. About 15 articles examined cancer-related policies and financing mechanisms. Policies, though promising, showed mixed progress. The NHIF reached only 9%–17% of Kenyan population with partially low enrollment among the informal sector workers (27%), which constitutes majority of working population. National health financing stood at 3.5% of GDP, below the WHO’s 6% benchmark [11, 16, 62]. This chronic underfunding severely constrains the system’s capacity to absorb the rapidly rising costs of cancer care. There was limited data (only 2 articles) on the post-2023 SHA reforms limiting possibility of robust evaluation.

Despite this, the scoping review identifies several potentially transformative mechanisms within the SHA framework, providing a foundational synthesis of the available evidence. These include ECCIF and the proposed oncology benefit package initially set at KES 400,000, with discussions of expansion. If implemented effectively, it could meaningfully reduce treatment-related financial hardship. However, infrastructure gaps, including workforce shortages, low uptake and weak procurement governance, dilute its potential impact [10, 46, 62]

Procurement delays were repeatedly reported. These delays contribute directly to medicine stockouts and force patients to seek care in private facilities at higher costs [9]. Decentralisation has increased chemotherapy access (69.1%) and palliative care (57.9%), but radiotherapy remains domiciled in urban-centers restricting access for rural populations [39].Weak cancer registries; 64% missing staging data undermine evidence-based policy responsiveness and resource allocation. Patient distrust in NHIF due to inadequate reimbursement, with 44.9% reporting receiving less than expected, reflected perceived gaps between policy promises and real world benefits [4].Overall, these findings suggest that financial protection policies have expanded coverage but have not yet translated into consistent financial risk reduction for cancer patients, consistent with LMIC policy challenges [78]

The review had strengths and some limitations. Adherence to PRISMA-ScR guidelines, OSF registration, MMAT appraisal and grey literature inclusion, enhanced transparency and contextual relevance. The synthesis integrated clinical, economic and policy evidence, providing a multidimensional perspective on cancer medicine access in Kenya. Methodological inconsistencies in cost reporting also constrained cross study comparisons. Exclusion of databases (Embase, Medline, Scopus) due to access limitations may have introduced selection bias. Some international studies with Kenyan sub-analyses may therefore have been missed. Finally, there was sparse evidence on post 2023 SHA reforms, given the recent implementation. Nonetheless, since we identified 60 articles, it suggests that the mapped literature provides a meaningful overview of the current evidence landscape on cancer medicines access, FT, QoL and health policy performance in Kenya.

### Evidence before this study

Globally, FT is recognised as a major barrier to cancer care even in high-income countries with UHC. Studies report CHE in 13%–68% of households, while public sector medicine availability of cancer medicine in East Africa is at less than 50%. LMIC reviews on cancer care have laid a thoughtful foundation. The body of evidence, though varied, highlights systemic weaknesses; low insurance penetration, procurement inefficiencies, as issues of significance in cancer treatment access. In addition, the current body of evidence shows diverse methodologies in cost reporting for cancer medicines, suggesting the need for country-specific studies to highlight the cancer treatment access landscape.

### Added value of this study

To the best of our knowledge, this is the first scoping review to consolidate evidence on cancer medicine access, FT, QoL and health policy performance in Kenya. Current studies are fragmented, with inconsistent methods and limited literature on access, especially for colorectal and esophageal cancers. Nevertheless, this review demonstrates that Kenya’s cancer treatment access crisis is not due to medical scarcity but policy misalignment, where high costs, fragmented coverage and inefficient procurement perpetuate inequity. The review demonstrates that no studies have directly compared health insurance transition (NHIF to SHA) on cancer treatment outcomes in Kenya. It also highlights that FT and QoL validated tools are underused in Kenya, demonstrating the urgent need for more exploration using standardised methodologies, longitudinal data and policy-focused research to inform Kenya’s National Cancer Control Strategy.

### Implications of all the available evidence

High treatment costs, procurement inefficiencies, and inadequate insurance coverage disadvantage cancer treatment access in Kenya. Corrective strategies must integrate price regulation, pooled regional procurement and expanded SHA coverage to reduce FT and improve QoL. There is a need to strengthen cancer registries and adapt and harmonise validated patient-reported outcomes tools, such as the COST questionnaire for FT and the EORTC QLQ-C30 for QoL, to better capture the undetected financial burden and QoL impacts. Longitudinal and multi-stakeholder studies (patients, carers, providers and policymakers) using standardised measurement tools are needed to capture FT and QoL trajectories over time, given the rising cancer cases. Furthermore, policymakers should prioritise prospective evaluation of SHA reforms, as well as funding and training for researchers in health services research, to enhance the development of local expertise in cost-effectiveness analysis and financial protection initiatives for cancer patients.

## Conflicts of interest

I/we declare no conflicts of interest.

## Funding

The scoping review is a preliminary investigation and part of a larger study funded by National Cancer Research Fund (NRF), (NCI-NRF001/2024), Kenya. However, the funder had no role in study design, data collection, data analysis, data interpretation or writing of the report.

## Data sharing

The data used are available upon reasonable request.

## Author contributions

JOO, DOO and SAA conceived and designed the scoping review. JOO and SAA conducted study selection and data extraction. All authors contributed to data interpretation and analysis. JOO drafted the manuscript, with revisions and supervisory support from SAA and DOO. All authors read and approved the final manuscript.

## Supplementary information

Supplementary data are available, including full search strategy for PubMed, detailed inclusion and exclusion criteria (based on PCC framework) and data charting template (Excel format).

## Figures and Tables

**Supplementary Table S1.** PubMed pilot search strategy for studies on the five cancers.

Date	Database	Key words
31-May-25	PubMed	Search: (("Breast Neoplasms"[Mesh] OR "breast cancer"[Title/Abstract] OR "breast neoplasms"[Title/Abstract] OR "breast tumor"[Title/Abstract] OR "breast tumour"[Title/Abstract]) OR ("Uterine Cervical Neoplasms"[Mesh] OR "cervical cancer"[Title/Abstract] OR "cervix cancer"[Title/Abstract] OR "uterine cervical neoplasms"[Title/Abstract] OR "cervical neoplasms"[Title/Abstract]) OR ("Prostatic Neoplasms"[Mesh] OR "prostate cancer"[Title/Abstract] OR "prostatic neoplasms"[Title/Abstract] OR "prostate tumor"[Title/Abstract] OR "prostate tumour"[Title/Abstract]) OR ("Colorectal Neoplasms"[Mesh] OR "colorectal cancer"[Title/Abstract] OR "colon cancer"[Title/Abstract] OR "rectal cancer"[Title/Abstract] OR "colorectal tumor"[Title/Abstract] OR "colorectal tumour"[Title/Abstract]) OR ("Esophageal Neoplasms"[Mesh] OR "esophageal cancer"[Title/Abstract] OR "oesophageal cancer"[Title/Abstract] OR "esophageal neoplasms"[Title/Abstract] OR "esophageal tumor"[Title/Abstract] OR "esophageal tumour"[Title/Abstract])) AND ("Adult"[Mesh] NOT ("Child"[Mesh] OR "Infant"[Mesh] OR "Adolescent"[Mesh])) AND ("Kenya"[Mesh] OR Kenya[Title/Abstract]) AND (("Health Services Accessibility"[Mesh] OR "access to medicines"[Title/Abstract] OR "medicine availability"[Title/Abstract] OR "medicine affordability"[Title/Abstract] OR "Drug Costs"[Mesh] OR "Pharmaceutical Preparations"[Mesh] OR affordability[Title/Abstract] OR availability[Title/Abstract]) OR ("FT"[Title/Abstract] OR "Health Expenditures"[Mesh] OR " CHE"[Title/Abstract] OR "OOPcosts"[Title/Abstract] OR "Financial Stress"[Mesh]) OR ("QoL "[Mesh] OR "QoL "[Title/Abstract] OR QoL[Title/Abstract]) OR ("Health Policy"[Mesh] OR "health policy"[Title/Abstract] OR "Insurance, Health"[Mesh] OR NHIF[Title/Abstract] OR "NHIF"[Title/Abstract] OR SHIF[Title/Abstract] OR "Social Health Insurance Fund"[Title/Abstract] OR UHC[Title/Abstract] OR " UHC "[Title/Abstract])) AND ("2018/01/01"[PDAT] : "2025/08/31"[PDAT]) AND English[lang]

**Supplementary Table S2.** Eligibility criteria.

Criteria	Description	Rationale
Population	Adult cancer patients (>18 years) with breast, cervical, prostate, colorectal, or esophageal cancers in Kenya.	Focus on prevalent cancers with robust data; pediatric cancers excluded due to distinct pathways.
Concept	Studies addressing: 1) access to essential cancer medicines (prices, availability, affordability); 2) FT (OOP costs, CHE); 3) QoL (e.g., EORTC QLQ-C30, patient experiences); 4) health policy effectiveness (e.g., NHIF, SHIF, UHC).	Captures critical dimensions of cancer care barriers and policy impacts.
Context	Studies conducted in Kenya (public/private hospitals, community settings).	Ensures relevance to Kenyan healthcare system.
Inclusion	Primary research, policy evaluations; quantitative, qualitative, or mixed-methods; English; published January 2018–May 2025.	To align with UHC and NHIF/SHIF reforms.
Exclusion	Non-Kenyan studies, editorials, conference abstracts without full text, non-cancer populations.	To maintain focus and methodological feasibility.

**Supplementary Table S3.** Data items.

Variable	Description
Study characteristics	Author, year, study design, location, publication type (journal article/grey).
Population	Cancer type, demographics (age, sex), sample size.
Access to medicines	Prices (e.g., cost per dose), availability (e.g., stockout rates), affordability (e.g., cost-to-income ratio).
FT	OOP costs, CHE (>40% household income), coping strategies (e.g., borrowing, selling assets).
QoL	QoL scores (e.g., EORTC QLQ-C30), qualitative themes (e.g., psychosocial stress, stigma).
Policy effectiveness	Policies evaluated (NHIF, SHIF), outcomes (e.g., coverage rates), barriers (e.g., reimbursement delays).

**Supplementary Table S4.** Summary of cancer medicine affordability (days of minimum wage).

1 cycle of chemotherapy treatment	KNH (Days)	Cheapest private hospital (Days)	KEMSA (Days)	Cheapest supplier (Days)
Doxorubicin/Cyclophosphamide (HER2 negative)	5.51	9.69	3.15	3.72
Cyclophosphamide then Paclitaxel/Trastuzumab (HER2 positive)	162.42	111.84	144.91	78.36
Trastuzumab (Biological Therapy)	151.74	87.33	137.79	69.67
Docetaxel	10.28	9.34	-	9.73

**Supplementary Table S5.** Summary of findings on FT among cancer patients in Kenya.

Study	OOP expenditures	Catastrophic spending	Coping mechanisms
[16]	Medication (87%), travel (84%), diagnostics (75%)	High burden; many sold assets, borrowed, or stopped treatment	Help from relatives (10%), church (10%), charity (13%)
[4]	91.2% reported household financial impact; 44.9% reported inadequate insurance reimbursement	Not reported	53.8% of breast cancer patients forwent care due to cost;
[8]	Average treatment cost: KES 143,132; medicines and inpatient admissions as major cost drivers	Not reported	Not reported

[33]	$100–$300 for diagnostics; $85–$1,500 for treatment	Not reported	Not reported
[38]	$1,500–$4,000/year (public), $2,500–$7,500/year (private)	20.27% experienced catastrophic spending	Not reported
[43]	High cost of care; transportation costs	54% faced costs >40% household expenditure	Borrowing (81%), selling assets (73%)
[63]	High OOP for visits, medications, diagnostics; treatment costs drain household resources	Catastrophic expenditure reported; financial ruin to families	Selling assets, borrowing, fundraising
[44]	High OOP for Trastuzumab (KES 39,900/month), travel, and lost income	Not reported	External charity funding
[47]	Not reported	38% faced catastrophic expenditure	16% avoided care due to costs
[52]	High OOP for medications and procedures	Catastrophic spending related to poverty	Not reported
[10]	High OOP costs (19.9% of CHE, KSh 108B in 2020/21)	High burden; 50% with cancer avoid care due to cost	Not reported

Note: KES = Kenyan Shillings; $ = US Dollars

**Supplementary Table S6.** QoL of cancer patients.

Study	Cancer type(s)	QoL tools used	Quantitative QoL findings	Qualitative QoL themes
[17]	Multiple (not specified)	CIDI, 30-Day Functioning and Disability Scale, Family Burden Scale, Suicidality Scale, IES-R, BDI-II, BHS	Depression: 43.9% (mild/moderate/severe); PTSD: 44.2% (severe); Suicidality: 6% lifetime prevalence; Reduced social/occupational functioning	Reduced social life; Psychological distress (depression, PTSD); Mobility and self-care difficulties
[18]	Metastatic breast cancer	FACT-B	90.4% reported moderate to high unmet needs (psychological: 63%, physical/daily living: 60.5%, health system: 55.4%); Better QoL with urban residence, internet access, stable disease	Psychological distress; Spiritual needs; Healthcare system needs; Physical limitations; Information needs
[20]	Cervical cancer	EORTC QLQ-C30, EORTC QLQ-CX24	Global health status: 41.99 ± 31.4; 69% had poor HRQoL; Early-stage disease (AOR = 7.3, 95% CI = 2.4–21.7, *p* < 0.0001) and no comorbidities (COR = 3.1, 95% CI = 1.1–9.1, *p* = 0.037) predict better QoL	Emotional and social functioning deficits; Financial difficulties
[22]	Breast, prostate, lymphoma	EORTC QLQ-C30	Good QoL: 64% (breast), 85% (prostate), 58% (lymphoma); Partial remission: 45.5% (breast), 45.1% (prostate), 42% (lymphoma)	Not reported
[24]	Breast	Hamilton Depression Rating Scale (HAM-D)	Depression prevalence: 59.4%; Late-stage cancer (OR = 1.61, *p* = 0.319), employment (OR = 3.7, *p* = 0.058), chemotherapy (neoadjuvant OR = 9.43, palliative OR = 9.5, *p* < 0.05) associated with depression	Weakness; dry mouth; fatigue; insomnia; hypersomnia
[30]	Breast, prostate, kaposi sarcoma, lung, colon, esophagus, pancreas, rectum	EORTC QLQ-C30 (version 3.0)	Global health/QoL score: 53 ± 27; Social functioning: 51 ± 36; Financial difficulties: 79 ± 31; Fatigue: 56%; Pain: 65%	Social functioning lowest; financial difficulties highest
[31]	Breast	None (qualitative)	Not reported	Physical: Treatment side-effects (pain, fatigue); Social: Family support, stigma; Psychological: Depression, anxiety, negative body image; Spiritual: Religion as hope
[34]	Breast, cervical, others	Functional evaluation of chronic illness therapy	Not quantified; Low stigma levels (Awkwardness: 2.51 ± 0.75; Severity: 3.22 ± 1.29; Financial discrimination: 2.77 ± 1.17; Personal responsibility: 1.9 ± 1.38; Avoidance: 1.98 ± 0.60; Policy-level: 2.99 ± 1.17)	Emotional, financial, physical long-term impacts; Importance of nutrition; Social stigma

**Notes: QoL tools used:** EORTC QLQ-C30, FACT-G, FACT-Cx, FACT-E, NCCN Distress Thermometer, Hamilton Depression Rating Scale (HAM-D), Beck Anxiety Inventory (BAI), Beck Depression Inventory-II (BDI-II), CIDI, IES-R, BHS, FSFI, Body Image Scale, MSPSS, MVQOL-I, and Functional Evaluation of Chronic Illness Therapy. **Qualitative Themes:** psychosocial stress, stigma, financial burden, physical symptoms (fatigue, pain, nausea), body image distress, social isolation, spousal abandonment fears, and spiritual distress. Patient education and family support improve coping

**Supplementary Table S7.** Policy interventions and implementation barriers in cancer care in Kenya.

Policy/Program	Studies	Outcomes	Barriers
NHIF (pre-2023)	[4, 10, 11, 16, 31–33, 50, 62]	Limited coverage (9%–17%); covers inpatient bed costs only; 27% informal sector uptake; high OOP costs; limited impact on treatment affordability; inequitable coverage (4.1% poorest versus 57.2% richest); covering only 4 of 8 chemotherapy cycles	Low informal sector enrollment (27%); inpatient-only benefits; non-comprehensive coverage (excludes treatment/diagnostics); unaffordable premiums (KES 500/month); inequitable access; inadequate reimbursement (44.9% received less than expected); delayed payments
SHIF (Post 2023)	[62, 67]	Potential OOP cost reduction via mandatory contributions (2.75% income-based), and KES 400,000 cap. ECCIF covers oncology services (such as chemotherapy KES 5,000/session, 1st line treatment limit KES 400,000 SHIF, KES 250,000 ECCIF)	Limited infrastructure; workforce shortages; funding gaps; reliance on empaneled facilities
NCCS (2023–2027)	[10, 11, 36, 61, 62]	Legal frameworks established (e.g., NCI-K, county committees); proposed hub-and-spoke model; Cancer Fund operationalization	Inadequate financing (3.5% GDP on health, below WHO’s 6%); weak cancer registry (64% missing staging data); limited human resources; urban service concentration; poor policy implementation; weak coordination
Cancer Prevention and Control Act 2012	[10, 11, 38]	Established legal frameworks; progress in NCI-K operationalization	Limited financing; gaps in implementation; no Cancer Act regulations; insufficient funding
Kenya Cancer Policy 2019–2030	[10, 53]	Framework for increased budget allocation, expanded NHIF coverage, improved infrastructure; aims to enhance social support	Limited costing data; incomplete cancer registry data; insufficient funding; weak enforcement of regulations
National Cancer Screening Guidelines	[57]	Low service availability (26% screening, 2% treatment, 3% palliative care)	Inadequate facilities; lack of equipment/staff; urban bias; low awareness (<50% for cervical screening)
National Guidelines for Cancer Management 2013	[11, 58]	Standardized protocols for diagnosis/treatment/care	Late diagnosis; limited facilities; inadequate human resources; lack of cost data; no implementation evaluation
Kenya National Palliative Care Guidelines 2013	[48]	Not reported	Inadequate pain management; limited healthcare providers
KEML 2023	[66]	Guides procurement and UHC packages; antineoplastics at levels 4–6	Human resource/infrastructure capacity limits; potential fund diversion to high-cost drugs; PPB registration reliance
Kenya Ministry of Health Guidelines	[33]	Increased clinically appropriate care (39.5% versus 8%) after positive cervical cancer screen; improved data quality	Limited provider capacity; poor linkage between screening/treatment points; patient travel/treatment costs; incomplete paper registries
Health Financing Strategy 2016–2030	[32]	Limited NHIF impact; aims to reduce OOP reliance	Lack of strategic leadership; inconsistent stakeholder priorities; inefficient population identification; limited county financial support
Social Health Insurance Act 2023	[54]	Establishes mandatory SHIF registration; ECCIF for cancer treatment	Limited infrastructure; workforce shortages; funding gaps
ECCIF￼	[62]	Potential to mitigate catastrophic spending for cancer	Limited infrastructure; workforce shortages; funding gaps
Kenya Health Policy 2014–2030	[38, 59]	Aims to reduce OOP reliance; expand UHC	High treatment costs; low awareness; poor health-seeking behavior; inadequate facilities; insufficient cancer policies
Health Sector Strategic Plan 2018–2023	[59]	Not reported	Inadequate quantification; lack of pharmacists; insufficient funding; urban bias; poor prioritization
Supply Chain Strategy 2020–2025	[59]	Not reported	Inadequate quantification; lack of pharmacists; insufficient funding; urban bias; poor prioritization
National Cancer Registry	[33]	Not reported	Incomplete paper registries
KILELE Health Association Survivorship Program	[34]	Low stigma levels (e.g., awkwardness: 2.51 ± 0.75; financial discrimination: 2.77 ± 1.17); high need for awareness and advocacy	Lack of awareness; policy-level prioritization
Kenya Metastatic Breast Cancer Network	[18]	High uptake of website (13,944 visits, 379,861 hits in 2 years); documented unmet needs	Rural residence; poor education; limited internet; systemic delays

**Notes: Studies** column lists all records from the document that explicitly mention the policy/program. **Outcomes** are summarized based on the specific findings reported in the document for each policy/program. **Barriers** are directly taken from the document’s reported barriers to policy implementation; some policies have limited or no -reported outcomes/barriers in the document as noted

**Table 1.** MMAT table for cancer medicines, FT, QoL and health policy studies.

Screening questions	Yes	No	Can’t Tell	Total
S1. Are there clear research questions?	47	0	0	47
S2. Do the collected data allow to address the research questions?	47	0	0	47
**1. Qualitative studies**				
1.1. Is the qualitative approach appropriate to answer the research question?	14	4	1	19
1.2. Are the qualitative data collection methods adequate to address the research question?	14	4	1	19
1.3. Are the findings adequately derived from the data?	14	4	1	19
1.4. Is the interpretation of results sufficiently substantiated by data?	15	4	0	19
1.5. Is there coherence between qualitative data sources, collection, analysis and interpretation?	14	4	1	19
**4. Quantitative descriptive studies**				
4.1. Is the sampling strategy relevant to address the research question?	31	0	0	31
4.2. Is the sample representative of the target population?	21	6	4	31
4.3. Are the measurements appropriate?	31	0	0	31
4.4. Is the risk of nonresponse bias low?	25	6	2	32
4.5. Is the statistical analysis appropriate to answer the research question?	30	2	0	32
**5. Mixed methods studies**				
5.1. Is there an adequate rationale for using a mixed methods design to address the research question?	10	2	2	14
5.2. Are the different components of the study effectively integrated to answer the research question?	12	2	0	14
5.3. Are the outputs of the integration of qualitative and quantitative components adequately interpreted?	9	5	0	14
5.4. Are divergences and inconsistencies between quantitative and qualitative results adequately addressed?	8	6	0	14
5.5. Do the different components of the study adhere to the quality criteria of each tradition of the methods involved?	11	4	0	15

**Table 2.** Study characteristics.

Author(s) (Year)	Location	Study design	Cancer type(s)	Sample size	Outcomes measured	Summary of key findings
Owenga and Nyambedha (2018) [16]	Kisumu (JOOTRH)	Cross-sectional (Mixed-methods)	Cervical	334	FT, QoL, NHIF impact	Significant FT, very low insurance uptake (9% on NHIF), Fund covers inpatient services only, high OOP on medications, transport, diagnostics
Ndetei *et al* (2018) [17]	Nairobi (KNH)	Cross-sectional descriptive	Multiple (not specified)	389	QoL (psychological distress, depression, functioning)	Increasing cancer stage correlated with higher disability (*p* = 0.001), depression (*p* = 0.038) and reduced functioning, high prevalence of mental disorders
Shaikh *et al* (2022) [18]	Nairobi, Eldoret, Mombasa	Needs assessment survey + intervention	Metastatic breast cancer	114	QoL, unmet needs, intervention uptake	Psychological (63%), physical support (60.5%) and healthcare system (55.4%) needs highest unmet needs. Better QoL associated with urban residence, internet access and stable disease. Low breast cancer knowledge
Kibaara and Degu (2023) [19]	Nairobi (KNH)	Cross-sectional	Cervical	151	Adverse events prevalence	High prevalence (100%) of adverse events, ulcerated sores (52.8%) dysuria (7.5%) thrombocytopenia (5.6%) mostly probable (80.1%) per Naranjo scale, predominantly in radiotherapy patients (80.8%)
Shajahan Ahamed and Degu (2023) [20]	Nairobi (KNH)	Cross-sectional	Cervical	103	QoL, financial difficulties	69% poor HRQoL (mean global health score 41.99 SD = 31.4); early-stage disease patients 7.3 times more likely to have good HRQoL (AOR = 7.3 95% CI = 2.4–21.7 *p* = 0.000); patients with no comorbidities 3.1 times more likely to have good HRQoL (COR = 3.1 95% CI = 1.1–9.1 *p* = 0.037). Advanced disease stage and comorbidities predict poor HRQoL
Isaaka *et al* (2022) [21]	Nairobi (KNH)	Retrospective cross-sectional	Cervical	100	Treatment adverse effects (nephrotoxicity)	45% prevalence of cisplatin-induced nephrotoxicity (36% grade 1, 9% grade 2). Comorbidities (AOR = 8.4 *p* = 0.02) hypertension (AOR = 3.4 *p* = 0.02) and ≥3 cycles (AOR = 4.5 *p* = 0.027) significant risk factors
Degu *et al* (2022) [22]	Nairobi (KNH)	Prospective cohort	Breast, prostate, Lymphoma	231	QoL, mortality, remission rates	Mortality rates: 3% (breast), 4.9% (prostate), 10% (lymphoma); Most patients had partial remission (45.5% breast, 45.1% prostate, 42% lymphoma); Good overall health-related QoL (64% breast, 85% prostate, 58% lymphoma); Predictors of mortality: age >60, comorbidities, distant metastasis, advanced stage
Subramanian et al (2019) [4]￼	Multiple regions (Nairobi, Mombasa, countrywide)	Cross-sectional	Breast	800	FT, access barriers	Cost and transportation major barriers for both cohorts; 53.8% with breast cancer forwent care due to cost; 91.2% reported financial impact after cancer diagnosis, 44.9% reported inadequate insurance reimbursement for medical costs
Wambalaba *et al* (2019) [23]	Nine counties (Kakamega, Kisumu, Nakuru, Uasin Gishu, Baringo, Nairobi, Nyeri, Machakos, Mombasa)	Descriptive survey	Breast, cervical, esophageal, prostate, lymphoma, others	1,048	Access barriers, late-stage diagnosis	Most prevalent cancers: breast and cervical (women), esophageal and prostate (men); 80% cases diagnosed late at advanced stages; limited screening/treatment capacity, especially in rural areas; private facilities offer more services/ mostly in Nairobi; Private facilities offer more services than public
Saina *et al* (2021) [24]	Eldoret (MTRH)	Cross-sectional descriptive	Breast	79	QoL (depression prevalence)	59.4% prevalence of depression; late-stage cancer (OR: 1.61, *p* = 0.319), employment (OR: 3.7, *p* = 0.058) and chemotherapy (neoadjuvant OR: 9.43, palliative OR: 9.5, *p* < 0.05) significantly associated with depression
Kizub *et al* (2022) [2]	Kenya (nationwide)	Comparative analysis (quantitative)	Multiple (breast, cervical, esophageal, colorectal, prostate, others)	N/A	Medicine access, affordability	All WHO EML regimens unaffordable/ Generic cytotoxic affordable for governments; targeted therapies (e.g., Trastuzumab imatinib) unaffordable without 93%–99% price reductions; no regimens affordable OOP
Bosire *et al* (2020) [25]	Kenya (Nairobi)	Cross-sectional (Mixed-methods: quantitative questionnaire and qualitative FGD)	Breast and cervical	157 (quantitative) + 10 (FGD)	QoL, coping strategies	High prevalence of psychological effects: anxiety (79%), negative body image (65.6%), low self-esteem (63.1%), loneliness (55.4%), sadness (51.6%). Effects aggravated by low income (<20,000 KSH/month, 73.2% of participants) and more chemotherapy sessions (*r* = 0.51); age (*r* = −0.300) and marital status (married, *r* = −0.389) associated with fewer effects. FGD themes include psychological stress (body image, loneliness, emotional changes, cognitive effects) and coping (prayers, finding reason to live). Impacts treatment adherence.
Owenga (2018) ￼[26]	Kisumu (JOOTRH)	Cross-sectional descriptive	Cervical	334	QoL, stage presentation	Poor physical (60%) and functional (66%) well-being linked to late-stage presentation (Stage III/IV: 73%); fair overall QoL (57%); stage IV (54%) and III (19%) predominant; significant association between cancer stage and QoL (p < 0.0001)
Wang’Ombe and Kathungu (2021) [27]	Nairobi, Nyeri	Correlational	Not specified (various cancers)	96	QoL (pain, weight loss, sleep)	Low recovery outcomes: Mean 47.0 (SD 9.465); Pain: High pain (32.9%); Weight: Loss (80.5%); Sleep: Poor (57.3%); QoL: Poor (56.1%)
Obora *et al* (2022) [28]	Nairobi (tertiary hospital)	Cross-sectional	Gynecological (cervical, endometrial, ovarian, vulvar, vaginal)	108	QoL (sexual dysfunction, body image, social support)	85% sexual dysfunction; lubrication most affected (mean 0.91) aOR = 0.05) stage 3 (aOR = 9.81) low social support (aOR = 1.29) predict dysfunction
Makau-Barasa *et al* (2020) [11]	Nationwide (Nairobi, Eldoret, Mombasa, Nakuru, Nyeri, Kisii)	Policy reviews with qualitative components	All cancers (emphasis on cervical, breast, prostate)	14	Policy barriers, stakeholder roles	Policies established legal and implementation frameworks; Gaps in financing, human resources, decentralisation; Key informant survey identified barriers: high costs, stigma, poor communication, centralised services; Stakeholder analysis highlighted roles of government, NGOs, private sector, academia, media, international groups
Atieno *et al* (2018) [8]	Nairobi (KNH)	Cross-sectional cost-of-illness	Cervical, breast, prostate, esophageal, others	412	FT, treatment costs	High treatment costs (avg. KES 143132); medicines and inpatient admission are major cost drivers; higher costs in private sector
Ezzi *et al* (2019) [29]	Nairobi (KNH)	Cross-sectional	Genitourinary, gastrointestinal, head and neck, breast, musculoskeletal, lung	67	Treatment adverse effects (neuropathy)	83.6% prevalence of cisplatin-induced peripheral neuropathy (CIPN); 81% mild (grades 1–2); 3.6% grade 4; overweight/obese patients nearly all developed CIPN (not statistically significant)
Davda *et al* (2021) [30]	Eldoret (MTRH)	Cross-sectional	Breast, prostate, kaposi sarcoma, lung, colon, esophagus, pancreas, rectum	100	QoL, financial difficulties	Global health/QOL score 53 ± 27; functional scores 51–68; symptom scores 12–79
Annamalai *et al* (2024) [31]	Nairobi (AKUHN)	Qualitative (mixed-methods pilot)	Breast	40	QoL, FT	High prevalence of stress, anxiety, depression; negative impacts on mental health, QoL; financial burdens, spousal relationship strain
Oraro-Lawrence and Wyss (20￼20) [32]	Nairobi, Kisumu	Qualitative (key informant interviews)	Not applicable (health financing focus)	13	Policy effectiveness, FT	Policy effectiveness, FT Unaffordable premiums and inadequate infrastructure hinder UHC; policy implementation gaps identified
Mabachi et al (2022) [33]	Rift Valley (provincial hospital)	Observational with historical controls	Cervical	2,193	FT, care quality	Barriers to cervical screening: poor access, lack of awareness, socio-cultural influences
Maureen *et al* (2024) [34]	Nairobi County	Cross-sectional mixed-methods	Breast, cervical, colorectal leukaemia, nasopharyngeal)	42	FT, QoL, stigma	High FT /discrimination, reduced QoL, stigma among patients
Kamau *et al* (2024) [35]	Eldoret (MTRH, Alexandria Equra)	Longitudinal	Esophageal	59	QoL, treatment outcomes	Specific QoL indicators identified as prognostic factors; baseline HRQoL mean 107.1 (compromised QoL); post-treatment improvement with chemotherapy + surgery (*p* = 0.04), deterioration with radiotherapy alone (*p* = 0.0092); baseline HRQoL significantly associated with post-treatment HRQoL (*p* = 0.0065).
Mchidi *et al* (2024) [36]	Nairobi, Mombasa	Qualitative cross-sectional	Not specified	Not specified	FT, access barriers	Financial burden, late-stage diagnosis, cultural taboos, low referral rates
Mushani *et al* (2024) [37]	Nairobi	Qualitative longitudinal	Breast, Cervical	18	QoL, side effect management	Patients silently endure post-treatment symptoms (fatigue, alopecia, skin and nail changes); need for culturally relevant education
Douglas (2021) [38]	Nationwide	Quantitative cross-sectional	Cancer (unspecified), hypertension, diabetes	37,500	FT, catastrophic spending	CHE due to cancer significantly increases household poverty; education and urban locality reduce poverty
Nyangasi *et al* (2023) [39]	12 Regional Cancer Centres + National Referral Hospital	Descriptive (secondary data analysis)	Not specified (various cancers)	321	Access, stage presentation, policy outcomes	Low service availability, urban concentration, 70%–80% late diagnoses, >50% paediatric abandonment
Nungo*et al* (2024) [12]	Kenya (Nationwide focus on NHIF)	Retrospective policy analysis (mixed-methods: interviews and document analysis)	Not applicable (focus on health financing, not specific to cancer)	21 interviews	Policy effectiveness, equity implications, informal sector participation, inefficiencies in purchasing/payment,	Low NHIF coverage, only 17% of Kenya’s population covered by SHI (as of 2023); 27% informal sector NHIF coverage; limited stakeholder engagement and expert advice adoption; political affiliations heavily influence policies; inefficiencies in purchasing/payment (e.g., slow reimbursements, misappropriations, favoritism); group schemes and penalties exacerbate inequity in access.
Daniel *et al* (2023) [40]￼	Nairobi (KNH)	Mixed-methods	Breast	378	FT diagnosis/treatment delays	Significant institutional delay in access to chemotherapy and radiotherapy due to healthcare system barriers
Orindi *et al* (2021) [41]	Kisii	Descriptive cross-sectional	Breast, cervical, prostate, leukaemia, others	120	QoL, social support	Pain relief and psychosocial counseling predominant; significant association between cancer type, treatment and QoL scores
Olwanda et al (2024) [42]	Kenya (national-level model)	Budget impact analysis (Markov model)	Cervical	N/A	Cost-effectiveness, QALYs	Population-based cervical cancer treatment costs $531,100 over 20 years, compared to $55,398 for ad hoc screening; systematic approach optimises resource utilisation, reduces unnecessary testing and lowers financial burden for patients and the healthcare system.
Sherman and Okungu (2018) [43]	Mombasa County	Descriptive mixed-methods (FGDs, interviews, questionnaire)	Breast	72	FT, access barriers, QoL	High cost of care; barriers include transportation, stigma, poor provider communication; poor QoL due to delayed/wrong diagnoses, surgical complications, equipment failures
Wabende *et al* (2022) [44]	Eldoret	Retrospective chart review	HER2-positive breast	95	Medicine access, treatment completion	Low availability of HER2-targeted therapies (e.g., trastuzumab); high treatment abandonment due to high costs; financial burden reduces QoL; need for policies to improve access to targeted therapies
Subramanian *et al* (2018) [45]	Kenya (Nairobi, Eldoret)	Cost analysis (itemisation cost approach)	Breast, cervical, prostate	N/A	FT, affordability	Substantial variation in patient costs between the public and private sectors. High OOP costs for diagnostics/treatment/travel; cost of care major barrier
Lehmann *et al* (2020) [46]	Nairobi, Nakuru, Kisumu, Kakamega, Kilifi, Siaya	Qualitative case study (FGDs, interviews)	Breast, prostate, cervical, esophageal, colon, others	32	FT, QoL, policy effectiveness	Unaffordable premiums, inadequate infrastructure, delayed payments, fraud; need to harmonise benefit packages
Toroitich *et al* (2022) [47]	Kenya (nationwide)	Critical literature review	Not specific (NCDs including cancer)	N/A	Medicine availability, affordability	Low medicine availability in primary facilities; urban bias in distribution
Justin Kinoti *et al* (2020) [48]	Machakos	Cross-sectional	Breast, cervical, esophageal, prostate, kaposi sarcoma	361	QoL, distress prevalence	Pain (83.3%), problem with decision making about treatment (64.9%), fatigue (59.8%). Other issues (financial constraints and eating difficulties). High distress prevalence; lower QoL in psychological domain
Ong'Ondi *et al* (￼2023) [49]	Nairobi (KNH)	Cross-sectional	Breast, cervical, gastrointestinal, prostate, others	361	QoL, distress prevalence	High distress prevalence; negative impact on QoL
Mutugi *et al* (2024) [3]	Nairobi (pharmacies)	Cross-sectional	Multiple (breast, prostate, others)	N/A	Medicine availability, affordability, pricing	Low availability of morphine; high OOP for medicines, availability highest in hospitals, followed by suppliers and finally supply agencies
Mbau et al (2023) [50]	Kenya (national, two counties)	Mixed methods (Embedded case study)	Multiple (oncology included)	41 interviews, 51 FGD participants	FT, policy effectiveness	Oncology services included in new packages but limited by infrastructure gaps; inequitable access due to pro-private facility distribution. Reforms aimed to expand coverage and reduce OOP but were hindered, requiring strategic alignment for UHC progress.
Angachi *et al* (2022) [51]	Eldoret, Kisumu (MTRH, JOOTRH)	Cross-sectional	Cervical	218	QoL (anxiety, depression prevalence)	High anxiety (80.3%) and depression (67%) prevalence; higher in 40–49 years (anxiety: 29.8% depression: 25.2%) primary education (anxiety: 46.8% depression: 42.2%) married (anxiety: 54.1% depression: 42.7%) and those with family support (anxiety: 44.5% depression: 36.2%); no significant associations (*p* > 0.05)
Mwarania (2022) [52]	Kenya	Cross-sectional (NHA, KHHEUS analysis)	Cancer (unspecified)	37,500	FT, catastrophic spending	CHE due to cancer significantly increases household poverty; education (OR = −0.2346 *p* = 0.012) and urban locality (OR = −2.2645 *p* = 0.000) reduce poverty; household size (OR = 0.0277 *p* = 0.001) increases poverty; gender (male OR = −0.0372 *p* = 0.374) not significant
MOH (2019) [53]	Nationwide	Policy analysis	All cancers	N/A	FT, policy barriers	Significant FT, OOP costs a major barrier to care; inadequate NHIF coverage limits evidence-based standards. Cancer medicine access hampered by stock-outs, complex procurement processes, and poor regulation of importation/quality/pricing; KEMSA's bulk procurement leverages economies of scale for better pricing, with calls for pooled mechanisms, local production, and Public Procurement Act amendments. Policy impacts include NHIF oncology package but with gaps in equity/efficiency; overall, aims for sustainable financing, better
Republic of ￼Kenya (2023) [54]	Nationwide	Regulatory document	All cancers	N/A	Oncology services scope	Details tariffs for oncology services
Njuguna et al (2022) [55]	Nationwide	Cross-sectional; Policy brief, (NHA, KHHEUS analysis)	Non-communicable diseases (including cancers)	Not specified	FT, policy coverage	OOP share of CHE decreased from 23.9% (2015/16) to 19.9% (2020/21), but absolute OOP increased 20.7% from KSh 90B to 108B; per capita health expenditure rose 35% from KSh 4,914 to 6,640; government financing at 52.3%, SHI at 12.5%; insurance coverage 25.9% but inequitable (4.1% poorest versus 57.2% richest); CHE incidence fell from 6.6% to 4.5%, impoverishment from 1.1% to 0.7%; SHI insufficient alone for UHC
MOH (2022) [10]	Nationwide	Mixed methods (scoping review, interviews, FGDs)	Breast, cervical, esophageal, prostate, colorectal	N/A	FT, policy implementation	Limited availability of essential cancer medicines, with only 44% of 24 tracer medicines in facilities; KEMSA's procurement faces delays (4–8 months), High OOP costs (19.9% of CHE, KSh 108B in 2020/21) drive FT; 23% of health financing from OOP, NHIF covers only 17% of the population, with low informal sector uptake (27%), inefficient claims processing hinder UHC; Poor QoL due to advanced-stage diagnoses (70%–80% late-stage), inadequate palliative care, proposed hub-and-spoke model, Cancer Fund, and NCI-K operationalisation aim to improve access; policies advocate for price regulation, pooled procurement, and increased financing, but face challenges from poor coordination,
Gathecha Gladwell and Watiri (2022) [56]	Nationwide	Policy brief	All cancers (cervical noted)	N/A	FT, policy funding	Cancer-related OOP expenditure constitutes 19.9% of CHE (KSh 108B in 2020/21), with NCD spending at KSh 57.8B; NHIF covers only 17% of the population, with low informal sector uptake (27%), limiting financial protection for cancer care
Kagiri et al (2020) [57]￼	Nationwide	Cross-sectional survey (KHFA) with desk review	All cancers (cervical, breast, prostate, colorectal)	2,896 facilities	Medicine availability, access barriers	Limited availability of essential cancer medicines; only 15% of facilities have morphine for palliative care, Chemotherapy restricted to 11 public hospitals, High cost of cancer diagnosis and treatment contributes to significant financial impoverishment
MOH (2019) [58]	Nationwide	Guideline development	All cancers	N/A	Medicine availability, treatment protocols	Standardised protocols for diagnosis/treatment/care; limited cost data
Karumbi *et al* (2020) [59]	Nationwide	Cross-sectional survey /policy brief (KHFA) with desk review	All cancers (Palliative care focus)	2,980 facilities	Medicine availability, FT	Low availability, only 44% of 24 tracer essential medicines available in primary health facilities, with none having all 24. Morphine in 10% facilities; primary facilities 33%–50% versus 70% in hospitals;
Prince (2023) [60]	Kenya (Kisumu)	Qualitative (ethnographic fieldwork with therapeutic itineraries)	Cervical, endometrial	2 (focus on two middle-class women therapeutic itineraries, with broader data from other cancer patients)	FT, access barriers, QoL, precarity in private healthcare	Middle-class cancer patients face significant FT due to high OOP costs, reliance on private healthcare, and inadequate NHIF coverage. Prolonged treatment leads to catastrophic costs, asset depletion, and debt, exacerbating precarity.
Njuguna *et al* (2022) [61]	Kenya (National)	Cross-sectional, policy brief (NHA, KHHEUS analysis) Non-communicable diseases, including cancer)	Nationwide	N/A	FT, policy coverage	Total health expenditure (THE) increased to USD 4.9B; OOP at 24% (KSh 108B in 2020/21); government financing at 52.3%, SHI at 12.5%; insurance coverage at 25.9% but inequitable (4.1% poorest versus 57.2% richest); NCDs, including cancer, drive high OOP, necessitating sustainable domestic financing for UHC
Republic of Kenya (2024) [62]	Kenya (National)	Policy document / expenditure framework	General (includes cervical, colorectal, oncology services)	N/A	FT, policy effectiveness, medicine access	High OOP costs (24% of THE, KSh 108B in 2020/21) drive FT; cancer medicine access limited by stock-outs, inadequate KEMSA procurement, and urban bias; SHIF implementation (2.75% income-based contributions, min KES 300, max KES 5,000 monthly) reduces OOP for oncology services (e.g., chemo KES 5,000/session, radiotherapy KES 3,600/session); ECCIF supports chronic conditions; funding gaps persist, with only 17% NHIF coverage and low informal sector uptake (27%); recommends increased domestic financing and infrastructure investment to improve access and equity
Too and Lelei (2022) [63]	Kiambu County	Case study	Multiple (prostate, esophageal, cervical, cholangiocarcinoma, others)	12 caregivers	FT, QoL, policy effectiveness	High OOP costs; financial ruin to families; inadequate NHIF coverage with treatment costs draining household resources, leading to CHE and distress financing (DF)
Degu *et al* (2023) [64]	Kenya (Nairobi)	Cross sectional, retrospective cohort	Oesophageal cancer	299	disease progression, treatment response	Mortality rate 43.1%; 11.1% developed distant metastases; 20.1% disease progression despite treatment; 13.0% non-response; 1- and 5-year survival rates 86% and 25%; In advanced stages (III/IV), radiotherapy (AHR 3.3), chemotherapy (AHR 3.9), chemoradiation (AHR 5.6) significantly improved survival; Need for early detection and timely treatment to improve outcomes
Kung’U *et al* (2022) [65]	Kenya (Nairobi)	Descriptive cross sectional	Not specified (various cancers)	108	QoL, influencing factors	Age, cancer stage, time off treatment, education, and religious affiliation significantly predicts QoL; emphasises early detection, treatment, and spiritual support to improve QoL
MOH (2023) [66]	Kenya (National)	Guideline development	All cancers	N/A	Medicine availability, treatment protocols	KEML 2023 outlines essential cancer medicines aligned with WHO EML; includes chemotherapy, hormonal therapies (e.g., tamoxifen), and supportive care (e.g., morphine); aims to standardise treatment protocols and improve access through KEMSA
MOH (2023) [67]￼	Kenya (Nationwide)	Policy document/Tariff schedule	All cancers (emphasis on Breast, Cervical, Prostate, Colorectal, Childhood)	N/A	Policy effectiveness, FT	Details tariffs for oncology services under SHIF to reduce OOP; chemotherapy KES 5,000/session, 1st Line treatment – Limit of Kes. 400,000 from SHIF and KES. 250,000 from ECCIF, 2nd Line treatment – KES. 650,000 from ECCIF, aims to improve access and equity in cancer care
MOH (2023) [13]	Kenya (Nationwide)	Policy document / Strategy	All cancers	N/A	Policy effectiveness, FT, medicine access	Aims to reduce premature mortality by a third by 2,028 through prevention, early detection, diagnosis, treatment, palliative care; emphasizes UHC, equity, evidence-based interventions; discusses integration with NHIF/SHIF for financing, improving access to medicines via KEMSA procurement, addressing OOP costs, and enhancing QoL via survivorship and palliative care

**Notes: Sample size:** ‘N/A’ indicates studies without direct patient recruitment (e.g., policy analyses, facility surveys or model-based studies). **Setting**: Refers to the healthcare sector (public, private, faith-based, NGO) and specific facilities where applicable (e.g., KNH = Kenyatta National Hospital, MTRH = Moi Teaching and Referral Hospital, JOOTRH = Jaramogi Oginga Odinga Teaching and Referral Hospital, AKUHN = Aga Khan University Hospital Nairobi). **Outcomes Measured**: Aligned with the four objectives, including FT (OOP costs, catastrophic spending, coping strategies), QoL (quantitative tools, qualitative themes), medicine access/affordability and policy effectiveness

**Table 3.** Findings on prices, availability and affordability of cancer medicines in Kenya.

Study	Prices	Availability	Affordability
[45]	Public sector: $1,340–$1,543 for breast and cervical cancer stages I–III; Private: $7,500–$11,862	Not specified	High OOP costs; costs exceed average household expenditure ($413/annum); large proportion lack health insurance
[2]	All WHO EML regimens unaffordable OOP; no specific pricing provided	93.4% alignment with WHO EML; generic cytotoxics available, targeted therapies (e.g., Trastuzumab, Imatinib) limited	All regimens unaffordable; targeted therapies require 93%–99% price reductions
[4]	Not reported	Not reported; public and private sector access noted	53.8% of breast cancer patients forwent care due to cost; 91.2% reported household financial impact; 44.9% reported inadequate insurance reimbursement
[8]	Average treatment cost: KES 143,132; Chemotherapy: KES 6,000–600,000 per course	Not reported	High OOP costs; medicines and inpatient admissions are major cost drivers
[33]	Public sector: $180 for diagnostics, $85–$1,500 for treatment; Chemotherapy: $300 per course at KNH	Cryotherapy and LEEP available on-site; chemotherapy referred to KNH	High OOP costs: $100–$300 for diagnostics; cost barriers lead to forgoing care
[36]	Chemotherapy administration: KES 4,600, not covered by NHIF	Frequent stockouts, especially for chemotherapy	High costs due to drug prices and transport; limited NHIF coverage
[38]	Cancer treatment costs: $1,500–$4,000/year (public), $2,500–$7,500/year (private)	Not reported	20.27% of households experienced catastrophic spending; CHE increases household poverty
[44]	Trastuzumab: KES 39,900/month (USD 399, 1.33 doses at KES 30,000/unit)	Trastuzumab available but limited; only 33.4% completed 18 cycles	High OOP and travel costs; high treatment abandonment due to cost
[47]	Not reported	44% availability for 24 tracer medicines in public sector; none in primary facilities	38% faced CHE; 16% avoided care due to costs
[3]	Unit prices: KES 140 (Methotrexate 50 mg), KES 1,000 (Cisplatin 50 mg), KES 50,000 (Bevacizumab 400 mg), KES 86,432 (Trastuzumab 600 mg), KES 176,000 (Pembrolizumab 100 mg)	Hospitals: 50.8% (e.g., Carboplatin 78.9%, Trastuzumab 73.7%); Suppliers: 37.6%; Agencies: 29.4%; generics (e.g., Capecitabine 73.7%) more available than originators (6.7%)	All medicines unaffordable; chemotherapy cycles require 3.15–162.42 days of minimum wage (KES 411/day); e.g., Doxorubicin/Cyclophosphamide: 3.15–9.69 days; Paclitaxel/Trastuzumab: 78.36–162.42 days; Trastuzumab: 69.67–151.74 days; Docetaxel: 9.34–10.28 days
￼[10]	Not reported	Limited availability; only 44% of 24 tracer medicines in facilities; frequent stockouts	High OOP costs (19.9% of CHE, KSh 108B in 2020/21); 50% avoid care due to cost; limited NHIF coverage (17% population, 27% informal sector)
[59]	Not reported	Morphine: 5% in rural facilities, 50% in level 4, 100% in level 5/6; overall 44% for 24 tracer medicines (n = 2,980 facilities)	High OOP burden; 90% of medicine purchases OOP; forgoing care implied
[55]	Not reported	Not specified; implied limited due to high OOP	OOP at 24% of THE (KSh 108B in 2020/21); higher burden on lower wealth quintiles; insurance coverage inequitable (4.1% poorest versus 57.2% richest)
[67]	Chemotherapy: KES 5,000/session; Radiotherapy: KES 3,600/session; SHIF cap: KES 400,000 (USD 3,095) for treatment/diagnostics	Limited by stockouts, inadequate KEMSA procurement, urban bias	High OOP costs (24% of THE, KSh 108B in 2020/21); limited NHIF coverage (17% population, 27% informal sector); SHIF aims to reduce OOP

Note: KES = Kenyan Shillings; $ = US Dollars; N/S = Not Specified

**Table 4.** Policy implications for cancer care in Kenya from scoping review (2018–2025).

Policy implication theme	Description	Article IDs
Expand insurance coverage	Enhance NHIF/SHIF coverage to reduce OOP costs and FT, particularly for comprehensive cancer care and chronic disease management.	[4, 13, 16, 25, 60, 61, 63, 67]
Decentralise oncology services	Increase access to screening, treatment, and palliative care in rural areas to address urban-centric service bias.	[13, 23, 26]
Improve medicine procurement and availability	Strengthen KEMSA procurement systems, reduce stockouts, and promote local manufacturing or fast-track PPB registration for essential cancer medicines.	[2, 13, 66]
Subsidise costs and reduce financial barriers	Implement subsidies for diagnostics, treatment, and non-medical costs (e.g., transport) to improve affordability and reduce CHE	[4, 23, 67]
Enhance early detection and screening programs	Promote public awareness and expand access to early cancer screening to improve outcomes and QoL.	[13, 18, 20, 22, 23, 65]
Integrate psychosocial and QoL support	Incorporate routine psycho-oncological care, counseling, and spiritual support to address depression, stigma, and other QoL challenges.	[17, 18, 20, 24, 65]
Strengthen health infrastructure and workforce	Improve cancer care infrastructure, train more oncologists/pathologists, and address capacity limits to enhance service delivery.	[13, 23, 62]
Regulate pricing and private sector	Implement price regulation, differential pricing, and pooled procurement to reduce costs of cancer medicines and regulate extractive private medical markets.	[2, 60]
Enhance cancer registries and data systems	Strengthen cancer registries and health information systems to improve data quality for policy planning and resource allocation.	[13, 62]
Promote digital and community support	Expand digital platforms (e.g., Kenya Metastatic Breast Cancer Network) and community networks to provide education and psychological support.	[18]
Monitor and manage treatment adverse events	Enhance routine monitoring and management of adverse events (e.g., cisplatin-induced nephrotoxicity, radiotherapy side effects) to improve patient outcomes.	[19, 21]
